# Italian Oncology at the Crossroads: Between Hospital Bed Cuts and the Need for a Transition to Integrated Community-Based Simultaneous Care

**DOI:** 10.3390/cancers17111821

**Published:** 2025-05-29

**Authors:** Lavinia Gentile, Stefania Moramarco, Edoardo Carnevale, Fausto Ciccacci, Lorenzo Ippoliti, Giuseppe Liotta, Stefano Orlando, Giuseppe Quintavalle, Francesco Schittulli, Leonardo Palombi

**Affiliations:** 1 Department of Biomedicine and Prevention, University of Rome “Tor Vergata”, 00133 Rome, Italygiuseppe.liotta@uniroma2.it (G.L.); stefano.orlando@uniroma2.it (S.O.); leonardo.palombi@uniroma2.it (L.P.); 2 Faculty of Medicine, Saint Camillus International University of Health Sciences, 00131 Rome, Italy; 3 General Management ASL Roma 1, 00193 Rome, Italy; 4 Italian League for Fighting Against Cancer (LILT), 00161 Rome, Italy; 5 Catholic University of Our Lady of Good Counsel, 1026 Tirana, Albania

**Keywords:** length of stay (LOS), elderly patients, diagnosis-related groups (DRGs), cancer, infection, home care services, simultaneous care, hospitalization costs, delayed discharge, Italy

## Abstract

Background: In Italy, public health investments have not kept pace with the rising demand for cancer care. Hospitalization costs are increasing, and length of stay (LOS) remains a critical metric for hospital efficiency and care quality. Methods: An ecological study analyzed hospital discharge records of patients admitted to “Policlinico Tor Vergata” (Rome, Italy) in 2022. Associations between cancer types and key variables influencing inpatient care were analyzed using logistic regression models (AOR; 95% CI), along with discharge patterns. Results: Among 14,451 ordinary hospitalizations, cancer diagnoses accounted for 16.4%, with blood cancers as the largest subgroup (20.1%). LOS outliers (5%) contributed to 11,342 excess hospitalization days. Blood cancers were associated with prolonged LOS (2.031; 1.499–2.753), while blood (2.368; 1.911–2.933), gastric (2.216; 1.603–3.062), and bladder cancers (2.661; 2.133–3.319) had a higher infection risk. Patients with bladder cancers were more likely to be ≥65 years old (2.661; 2.133–3.319). Secondary diagnoses were more likely to occur in gastric cancer types (1.637; 1.486–1.802). A discharge analysis revealed that 46.8% of cancer patients were discharged home without activation of home care services, and only 0.2% received home care activation. Cancer patients were more likely to be discharged home (2.150; 1.911–2.418) while awaiting completion of diagnostic or therapeutic processes. Conclusions: Our findings highlight the significant variability in hospitalization patterns across cancer types and the inadequacy of current discharge planning processes. The burden of prolonged LOS highlights the unsustainability of current care models. An urgent transition toward integrated, community-based simultaneous care models is needed to reduce healthcare costs, prevent prolonged hospitalizations, and improve outcomes, particularly for vulnerable elderly patients.

## Simple Summary

Although there is increasing demand for oncology care in Italy, recent public health policies have resulted in the reduction of over a thousand oncology beds over the past decade. Cancers are often treated as a single entity; however, they encompass a diverse range of conditions that require tailored healthcare approaches. Given the significant burden associated with prolonged hospital length of stay (LOS), particularly for certain types of cancer, this study highlights the poor sustainable nature of current care models and underscores the critical importance of effective discharge planning for cancer patients. As a potential solution, this study supports the urgent need for a transition toward integrated community-based simultaneous care models to reduce healthcare costs and improve patient outcomes, especially for vulnerable elderly patients.

## 1. Introduction

Cancer represents a significant global health challenge, with its incidence rising largely due to shifts in age demographics. Despite being one of the leading causes of death worldwide, cancer is increasingly considered a curable disease [1]. Cancer patients are frequently admitted to hospitals throughout the course of their illness, also as a result of prolonged survival [2]. Hospitalization is typically considered unavoidable in most cases, as it is often the only way to ensure prompt diagnosis and treatment that cannot be managed at home or in long-term facilities [3].

The hospitalization of cancer patients is associated with substantial costs. A population-based cost analysis conducted across European Union countries found that hospital inpatient care accounted for more than half of cancer-related healthcare costs, although the economic cost varied by cancer type and between countries. Comparing the economic burden of different diseases is essential for policymakers and healthcare planners, as it informs decisions regarding resource allocation for service provision, prevention strategies, and research funding [4]. In Italy, hospitals face increasing demands due to rising cancer survival rates and the aging population—both factors expected to drive up healthcare needs. However, the planned National Health Fund (NHF) shows a notable year-on-year decline, with the funding allocated for 2024–2025 being substantially lower than the percentage of GDP that most European countries invest in their healthcare systems, at less than 6.5% of the GDP. Given that Italy has the highest proportion of elderly people in Europe, with over 24% of the population aged 65 or older [5], the health needs of the population might not be adequately covered by the Italian NHF in the coming years. This substantial underfunding of public health highlights a critical gap between available resources and patient care demands [6].

Length of stay (LOS) in hospitals is a key indicator of healthcare efficiency and plays a strategic role in hospital resource management [7,8]. Specific thresholds for hospitalization duration and rehospitalization rates have been proposed as quality indicators to enhance the effectiveness and sustainability of acute care services [9]. When LOS exceeds the predefined threshold based on Diagnosis-Related Groups (DRGs), the admission is classified as anomalous. Prolonged LOS is often linked to complications, such as infections [10], which significantly increase the demand for intensive care unit admissions [11].

A recent survey conducted across 98 Italian hospitals revealed that in 26.5% of cases, patients experienced delays of more than a week between their planned and actual discharge dates, leading to approximately 2.1 million excess hospitalization days. The elderly population has the highest hospitalization rates in the country, with 75.5% of these patients remaining hospitalized inappropriately due to a lack of family caregivers or available nursing home placements. Among those experiencing extended hospital stays, 64.3% were unable to access intermediate healthcare facilities in the community, while 22.4% faced challenges in accessing home care services [12,13].

Although cancers encompass a diverse range of conditions that require tailored healthcare approaches, they are often treated as a single entity [14].

We conducted an ecological study based on the complete dataset of adult hospitalizations recorded at the “Policlinico Tor Vergata”, Italy, over a one-year period (2022). The aim of this study was to provide a descriptive overview of the differences between patients with and without cancer, offering a snapshot of various cancer types and key variables that may influence cancer patient care, including infections, age, and prolonged length of stay (LOS). Being descriptive in nature and not based on a predefined hypothesis, this study seeks to support public policy by informing the allocation of government research funds and identifying potential alternative care models.

## 2. Materials and Methods

### 2.1. Sample

An observational retrospective study was conducted on ordinary admissions at “Policlinico Tor Vergata” (PTV), Rome, Italy, between 1 January 2022, and 31 December 2022. The facility is classified as a University Hospital (Azienda Ospedaliera Universitaria, AOU), meaning it is a healthcare institution integrated with the University of Rome “Tor Vergata”, and is involved in clinical care, research, and teaching activities. It has more than 580 inpatient beds with a covered area of approximately 140,000 square meters; it is located in an urban setting.

Data were retrieved from the administrative hospital discharge records (Scheda di Dimissione Ospedaliera, SDO), database of PTV, through the hospital’s central informatics system and provided by the Management Control Unit and the Hospital Information System of PTV, in compliance with applicable privacy regulations.

In accordance with the Italian privacy legislation (Law No. 2016/679) and Decree No. 85/2012 issued by the Guarantor for the Protection of Personal data, health data can be used in aggregate form and processed for epidemiological research purposes. All hospitalizations involving an overnight stay of at least one day were included in the study, while day-hospital and day-surgery admissions were excluded. For each admission, the following data were collected: age, gender, diagnosis, length of stay (LOS, in days), DRG-specific LOS threshold (in days), discharge ward, type of discharge, and outcome of admission. Diagnoses were classified according to the International Classification of Diseases, 9th revision, Clinical Modification (ICD-9-CM) and subsequently grouped into Diagnosis-Related Groups (DRG) Medicare version 24 [13].

To ensure privacy, all data were coded, with no personal identifiers included. Consent for data management, analysis, and publication was obtained from the Ethical Committee (Comitato Etico Indipendente—CEI) of the “Fondazione Policlinico Tor Vergata—PTV” (identification number 141.23, extraordinary meeting of 26 May 2023, held by the former Independent Ethics Committee, CEI). This study was conducted in compliance with the Ethical Principles for Medical Research Involving Human Subjects, as outlined in the World Medical Association Declaration of Helsinki (1975).

### 2.2. Statistical Analysis

Data were analyzed using the Statistical Package for the Social Sciences (SPSS, version 26, IBM, Somers, NY, USA) by the Hygiene and Preventive Medicine Department of the “University of Rome Tor Vergata”. Descriptive statistics (mean, median, standard deviation [SD], range, frequency, and percentage) were calculated for all variables.

Based on an SDO flow analysis, patients were classified as having cancer if at least one ICD-9-CM code related to cancer was recorded within the six diagnosis fields (one primary diagnosis and five secondary diagnoses). The results were presented both in total and stratified by cancer type. LOS was calculated from the day of hospital admission to the day of discharge or death.

Each Diagnosis-Related Group (DRG) has a predefined LOS threshold (in days), beyond which the admission is classified as anomalous. Hospitalizations exceeding this threshold were labeled as outliers, indicating a significantly prolonged LOS compared to other patients within the same DRG.

The statistical significance of differences between continuous variables was assessed using Student’s t-test, while categorical variables were analyzed using the chi-square test. LOS analysis was performed by cancer type, age group (cut-off at 65 years), and infection status (Odds Ratios and 95% Confidence Intervals: OR; 95% CI).

Binary logistic regression analysis was conducted to evaluate the relationships between cancer type and key variables of greater relevance from a public health perspective, particularly those affecting inpatient care (i.e., LOS outliers, infections, age ≥ 65 years, and secondary diagnosis) (Adjusted Odds Ratios and 95% Confidence Intervals: AOR; 95% CI). Specifically, the number of secondary diagnoses was used as a proxy for comorbidity burden across the different types of cancer based on the rationale that a greater number of secondary diagnoses recorded in the hospital discharge form reflects a higher burden of comorbidities. Given the presence of multiple independent variables, a stepwise forward selection method was employed. Additionally, the association between different discharge types in cancer-related hospitalizations was examined using both univariate and multivariate analyses to assess the probability of various discharge outcomes among cancer patients.

## 3. Results

A database was generated containing 21,735 hospitalizations recorded in 2022. After excluding one-day admissions such as day hospital and day surgery (7284 cases), a total of 14,451 ordinary hospitalizations for the adult population (18 years old) remained eligible for analysis.

When analyzing disease categories, cancer-related hospitalizations were the most frequent, with 2368 cases (16.4% of total ordinary admissions), followed by infections (2187 cases, 15.1%). Diabetes and dementia were also among the most common diagnoses, accounting for 1783 cases (12.3%) and 275 cases (2.0%), respectively.

Based on these findings, additional analyses were conducted, with a primary focus on cancer hospitalizations, as they represented the most frequent diagnosis. Table 1 presents the main characteristics of the study population, categorized by total hospital admissions (*n* = 14,451) and further stratified into cancer patients (*n* = 2368) and non-cancer patients (*n* = 12,083).

Out of the total ordinary hospital admissions, 56.8% were male and 43.2% were female. When examining the gender distribution between cancer and non-cancer patients, a significant difference was observed (*p* < 0.001), with males being more predominant in the non-cancer group, while females were more represented in the cancer group. The chi-square analysis indicated a highly significant association between gender and cancer prevalence (χ^2^(1) = 27.535, *p* < 0.0001), with a higher prevalence observed among females (18.2%) compared to males (15%) in our sample.

The overall sample had a mean age of 64.6 ± 16.6 years, with the mean age being slightly higher in the cancer group, though not statistically significant. Most patients were over 65 years old, with a higher proportion of cancer patients in the 65–75 age group (29.7% vs. 22.9%, *p* < 0.001), while non-cancer patients were more prevalent in the >85 age group (10.8% vs. 4.8%, *p* < 0.001).

Hospital admission type differed significantly (*p* < 0.001): non-cancer patients were more frequently admitted through the emergency department (63.1% vs. 38.1%), while cancer patients had more scheduled admissions (60.8% vs. 34.9%).

The mean length of stay (LOS) was 9.3 ± 13.1 days. A statistically significant difference was observed between groups, with cancer-related admissions having a longer LOS compared to non-cancer admissions (11.0 ± 13.2 days vs. 9.0 ± 13.0 days, *p* < 0.001). LOS outliers (hospitalizations exceeding DRG thresholds) contributed to 11,342 excess hospitalization days. These accounted for 5% of cases (*n* = 725; 127 cancer, 598 non-cancer patients), with no significant difference between groups. Among these, 65.1% were patients aged >65, totaling 6909 excess hospitalization days (61% of outlier days). For elderly cancer patients (>65 years), LOS outliers represented 53.5% of cancer-related outliers (846 excess days, mean LOS 12.4 ± 15.2 days), compared to 404 outliers among elderly non-cancer patients (mean LOS 15.0 ± 22.4 days).

**Figure 1:**
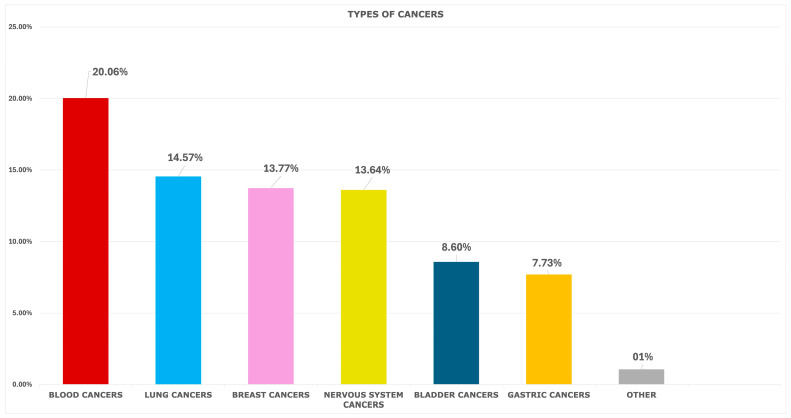
Cancer subtypes in cancer patients.

To assess the impact of different cancer types, patients with at least one cancer diagnosis were further categorized into subgroups based on affected organs and systems. Hepatopancreatic cancers (*n* = 487, 20.6%) and blood cancers (*n* = 475, 20.1%) emerged as the largest subgroups, together comprising over 40% of the total cancer cases. The second most prevalent type was lung cancer (*n* = 345, 14.6%), followed by breast cancer (*n* = 326, 13.8%) and nervous system cancers (*n* = 323, 13.6%). Bladder cancer accounted for 8.6% (*n* = 203), while gastric cancer represented 7.7% of cases (*n* = 183) (Figure 1).

The association between cancer type and key variables of greater relevance from a public health perspective (LOS outliers, infections, age ≥ 65 years, and secondary diagnosis) are presented in Table 2. Blood cancers were strongly associated with LOS outliers (AOR = 2.031, CI: 1.499–2.753, *p* < 0.001) and infections (AOR = 2.368, CI: 1.911–2.933, *p* < 0.001) but had a lower likelihood of affecting patients aged ≥65 years (AOR = 0.590, CI: 0.489–0.711, *p* < 0.001). In addition, patients with blood cancers displayed a significantly higher propensity for secondary diagnoses (AOR = 1.302, 95% CI: 1.176–1.858, *p* < 0.001). Breast cancer was less likely to affect older patients (≥65 years) (AOR = 0.516, CI: 0.412–0.648, *p* < 0.001) and had a lower risk of infection (AOR = 0.173, CI: 0.089–0.336, *p* < 0.001). Moreover, individuals with breast cancer were significantly less likely to present secondary diagnoses (AOR = 0.716, 95% CI: 0.662–0.773, *p* < 0.001). Nervous system cancers were linked to prolonged hospital stays (AOR = 1.940, CI: 1.255–2.998, *p* = 0.003), while a decreased risk of infection was observed (AOR = 0.517, CI: 0.348–0.768, *p* < 0.001). Patients were more likely to be under 65 years of age (AOR = 0.711, CI: 0.570–0.889, *p* = 0.003). These types of cancer were associated with a lower likelihood of secondary diagnoses (AOR = 0.906, 95% CI: 0.848–0.967, *p* = 0.003). Gastric cancer was significantly associated with infections (AOR = 2.216, CI: 1.603–3.062, *p* < 0.001) and older age (AOR = 1.673, CI: 1.216–2.301, *p* = 0.002). Furthermore, a strong positive association was observed between gastric cancer and the presence of secondary diagnoses (AOR = 1.637, 95% CI: 1.486–1.802, *p* < 0.001), consistent with the findings from the univariate analysis. A significant association was observed also between hepatopancreatic cancers and both LOS outliers (AOR = 0.460 CI: 0.258–0.820, *p* = 0.008) and age ≥ 65 (AOR = 1.241, CI: 1.032–1.494, *p* = 0.022). However, regarding secondary diagnoses, while the univariate analysis indicated a positive association, the multivariate model revealed a significant reduction in the likelihood of secondary diagnoses (AOR = 0.382, 95% CI: 0.213–0.686, *p* < 0.001). Lung cancer was strongly linked with older age in both models. There was no significant association between lung cancer and infections in the univariate model. No infection association was found in the univariate model, but a significant link emerged in the multivariate analysis (AOR = 1.862, CI: 1.476–2.348, *p* < 0.001; AOR = 0.689, CI: 0.495–0.959, *p* = 0.027). No significant associations were found between lung cancer and secondary diagnoses in either model. For bladder cancer, no association with LOS outliers was found, but significant associations with older age (AOR = 2.661, CI: 2.133–3.319, *p* < 0.001) and infections (AOR = 1.905, CI: 1.543–2.352, *p* < 0.001) were observed. As for secondary diagnoses, although a significant association was detected in the univariate analysis (OR = 1.129, *p* = 0.001), this effect was not confirmed after adjustment in the multivariate model.

Table 3 presents the number of discharges by type, length of stay (LOS) in days, and the results of univariate and multivariate analyses examining discharge probabilities in cancer-related hospitalizations.

**Figure 2:**
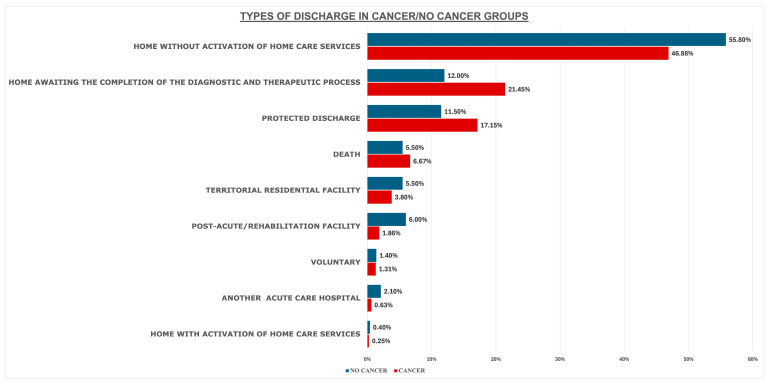
Types of discharge in cancer and non-cancer patients. Legend: home without activation of home care services: discharge to home without home care support after recovery; home awaiting the completion of the diagnostic and therapeutic process: patients staying at home while awaiting completion of diagnostic tests or treatment; protected discharge: discharge with follow-up care to ensure patient safety after leaving the hospital; death: death before discharge; territorial residential facility: long-term care facility for patients needing ongoing support in the local area; post-acute/rehabilitation facility: facility providing rehabilitation after serious illness, surgery, or injury; voluntary: discharge initiated by the patient or family based on personal choice; another acute care hospital: transfer to another acute care hospital; home with activation of home care service: discharge to home with pre-arranged home care support for recovery.

Figure 2 presents the analysis of discharge patterns. Discharges classified as “home without activation of home care services” were the most common type, accounting for 46.8% of cases. Conversely, discharges classified as home with home care activation were rare, occurring in only six cases (0.2%). For these patients, the recorded LOS was among the highest (34.3 ± 29.1 days), exceeding other discharge pathways.

The multivariate model analysis showed that cancer patients were more likely to be discharged home while awaiting completion of diagnostic/therapeutic processes (AOR = 2.150, CI: 1.911–2.418, *p* < 0.001), to be referred for protected discharge (AOR = 1.805, CI: 1.591–2.047, *p* < 0.001), or to die in the hospital (AOR = 1.472, CI: 1.225–1.768, *p* = 0.020). Conversely, they were less likely to be transferred to a post-acute/rehabilitation facility (AOR = 0.375, CI: 0.275–0.511, *p* < 0.001) or to another acute care hospital (AOR = 0.363 CI: 0.215–0.613, *p* < 0.001).

## 4. Discussion

The hospitalization pathway for cancer patients presents unique challenges, adding complexity to their hospital stays [2]. Although cancer encompasses a diverse range of conditions that require tailored healthcare approaches, it is often treated as a single entity [14]. In fact, certain cancer types, due to natural disease progression or treatment-related side effects, may require more frequent emergency visits. Conversely, for other cancer types, the clinical course increasingly resembles chronic disease management [3].

The primary aim of this ecological study was to provide a descriptive snapshot based on the complete dataset of adult ordinary hospitalizations recorded at the “Policlinico Tor Vergata” hospital in Italy over a one-year period (2022). The analysis offers an overview of the differences between patients with and without cancer from a public health–oriented perspective, focusing on selected factors known to affect inpatient care in ordinary hospitalizations, which may contribute to increased pressure on the National Health Service.

### 4.1. Summary of Key Findings

Our findings highlight distinct hospitalization pathways for different cancer types. Patients with blood cancers are more susceptible to infections, a condition that may further increase the need for extended hospital stays. This vulnerability reveals their clinical fragility, including immunocompromised states, which complicate discharge and transfer to other care settings. Their increased risk underscores the need for a well-structured home care system to support ongoing medical needs. A palliative and simultaneous continuum of care is essential, integrating a comprehensive home care framework. This approach bridges the gap between hospital care and recovery, improving patient outcomes and reducing complications in this high-risk population [15]. Our analysis confirms that nervous system cancers are more common in younger patients. While these patients have a lower probability of infections, they are at higher risk of prolonged hospital stays, aligning with previous studies that indicate that they often require complex postoperative care due to the nature of neurological surgeries and extended recovery periods [16]. For breast cancer patients, we observed an independent risk of prolonged hospitalization; however, this association disappeared in the multivariate model. This may reflect not only a lower severity of conditions but also recent socioeconomic investments in breast cancer care in Italy, particularly in the Lazio Region. In recent years, significant efforts have been made to enhance post-acute care and community-based management for breast cancer patients. Notably, the implementation of the Breast Cancer Care Pathway has led to a structured and comprehensive care system, improving post-hospitalization support and facilitating smoother transitions from hospital to community-based care, potentially reducing hospital stays [17].

Additionally, this study reinforces the well-established role of aging (≥65 years) as a major risk factor for some cancers, particularly lung cancer [18] and hepatopancreatic cancers. Older patients often present with advanced disease and complex treatment responses, leading to greater hospitalization needs [19,20].

Secondly, this study also aimed to assess essential healthcare services beyond hospitalization by analyzing discharge patterns. It is reasonable to suspect that prolonged hospital stays may not be solely driven by the clinical complexity of cancer but also by discharge difficulties stemming from inadequate community-based care. Despite being mandated by law, these services are often unevenly distributed and not universally accessible [21]. Severe deficiencies in integrated healthcare and social services—including day centers, home care, and simultaneous palliative care—may contribute to excess emergency department admissions and prolonged hospitalizations. In many cases, certain cancer types justify longer hospital stays due to the lack of alternative care services in the community [22]. This issue is likely to be particularly relevant to our findings, as our study was conducted in a disadvantaged area of Rome, where local healthcare services are insufficient. However, this can only be hypothesized as a contributing factor, since specific data on the socioeconomic conditions of the study population are unfortunately not available. Overall, our findings reveal a complex situation with integrated home care discharges applied in very few cancer cases, making it virtually irrelevant. This supports the hypothesis that limited community and home care services contribute to prolonged hospital stays beyond expected limits [23].

### 4.2. Implications of These Findings on Care Delivery

Studies confirm a strong association between prolonged length of stay (LOS) and increased healthcare costs [24], highlighting the urgent need for an effective network of community services, particularly in home-based integrated care [25]. In Italy, hospitalizations under the Diagnosis-Related Group (DRG) system accounted for EUR 26 billion in 2019 [12]. As specifically regards cancer, a previous study conducted in Italy in 2021 on one of the most significant cancers from a public health perspective, i.e., breast cancer, underscored the substantial economic strain imposed by this type of cancer. It revealed an annual financial burden—considering both direct healthcare costs and social security expenses—exceeding EUR one billion, including nearly EUR three hundred million annually for hospital admissions related to breast cancer in Italy. This study highlights the need for early detection and intervention strategies to mitigate costs and enhance patient outcomes [26]. Therefore, redefining the role of hospitals and ensuring better integration with territorial healthcare networks is crucial for improving efficiency and patient outcomes.

The positive impact of integrated care on hospital admissions and LOS is well-documented. Scientific evidence demonstrates that integrated care models, which emphasize community-based management, offer promising alternatives to hospital-centered approaches for cancer patients [27]. Hospital outcomes in integrated care models further support their effectiveness in reducing hospital stays for the general population [28,29], while also proving to be cost effective in preventing hospitalization and institutionalization, particularly for elderly populations [30,31].

These models have the potential to improve patient outcomes and optimize the use of healthcare resources. The available evidence supports established integrated care frameworks, such as those developed by the UK’s Local Government Association, which emphasize key principles including person-centeredness, coordinated service delivery, and strong partnerships between hospitals and community organizations. These elements are crucial for realizing the potential benefits of alternative oncology care models [32]. Furthermore, global analyses underscore that ongoing initiatives and innovations are progressively raising the standard of cancer care worldwide, reflecting a growing consensus on the need for comprehensive and equitable access to high-quality cancer services across healthcare systems [33].

### 4.3. The Way Forward

Simultaneous care can address unmet patient needs by integrating cancer treatment with radiotherapy, palliative care, psychological support, nutritional and integrative therapy, physical rehabilitation, holistic approaches, and smoking cessation programs. Its primary goal is to enhance the quality of life for patients and their families facing incurable diseases through prevention, early identification, and relief of suffering related to physical, psychosocial, and spiritual challenges. Currently, in Italy, simultaneous and palliative care represent the gold standard for providing optimal care to patients with advanced and/or metastatic cancer [34] even though official reports indicate that access to palliative care remains insufficient in the majority of Italian regions [35].

Additionally, the concept of a “virtual hospital”—capable of seamless interaction with community services and extending care beyond its physical boundaries—is now both feasible and cost effective through digital medicine. Highly efficient virtual wards can monitor patients at home via telemedicine, telemonitoring, and teleconsultation, alongside mobile units for urgent medical analyses or procedures [36]. Integrating these services with social and healthcare networks can reduce LOS, prevent unnecessary hospitalizations, and lower infection risks [37,38].

### 4.4. Limitations

This is an observational study, so findings should be interpreted with caution given the study design. A limitation of this study is that it focuses on a single hospital population; however, the authors plan to apply the same methodology in a future multicenter study. Secondly, comorbidities were investigated from a quantitative perspective (i.e., number of secondary diagnoses) rather than from a qualitative one (i.e., specific types of comorbidities). In addition to clinical conditions, we acknowledge that differences in treatment regimens and sociodemographic characteristics may also have contributed to the observed outcomes. Finally, it should be acknowledged that differences in discharge status among cancer types may reflect not only the actual intensity of post-discharge care needs but also the availability of healthcare services. Due to the absence of specific and reliable indicators within our dataset, we were unable to disentangle these two contributing factors, which represents a further limitation of the present study.

We acknowledge that since this study was conducted using 2022 data flow (1 January–31 December), the results might not have been influenced by the COVID-19 pandemic emergency. As a matter of fact, when we conducted the study, the official national data for 2022 were not yet fully available; preliminary reports indicate a gradual recovery in hospitalization rates, with volumes nearly returning to pre-COVID-19 levels [39].

## 5. Conclusions

Although integrated community-based simultaneous care for cancer patients is widely recognized and mandated by Italian legislation, its implementation remains insufficient. The lack or limited availability of these services contributes to longer, more frequent, and costlier hospital stays. In Italy, alongside the planned hospital bed cut, hospitals will continue to face rising demands due to increasing cancer survival rates and an aging population, both of which are expected to escalate healthcare needs. Therefore, it is crucial to rethink the healthcare system to better integrate hospital care with community- and home-based monitoring, control, and treatment.

## Figures and Tables

**Table 1:** Descriptive characteristics of the hospitalized population presented as overall ordinary admission and stratified by cancer and non-cancer patients.

Characteristics at Admissions	Total Ordinary Admissions *n* = 14,451	Non-Cancer Patients *n* = 12,083	Cancer Patients *n* =2368	*p*-Value
Gender, *n*. (%)				<0.001 *
Male	8212 (56.8%)	6982 (57.8%)	1230 (51.9%)	
Female	6239 (43.2%)	5101 (42.2%)	1138 (48.1%)	
Age				
Age range (min–max)	18–101	18–101	18–94	
Mean age (years ± SD)	64.6 ± 16.6	64.5 ± 17.2	65.2 ± 13.6	0.060 ^#^
Age ≤ 65 *n*. (%)	6395 (44.3%)	5369 (44.4%)	1026 (43.3%)	0.321 *
Age > 65 *n*. (%)	8056 (55.7%)	6714 (55.6%)	1342 (56.7%)	
Between 65 and 75 years	3474 (24%)	2771 (22.9%)	703 (29.7%)	0.001 *
Between 75 and 85 years	3251 (22.5%)	2725 (22.6%)	526 (22.2%)	0.718 *
Over 85 years	1331 (9.2%)	1218 (10.8%)	113 (4.8%)	0.001 *
Type of admission, *n*. (%)				0.001 *
Transfer from Another hospital	271 (1.9%)	244 (2.0%)	26 (1.1%)	
Emergency Department (ED)	8524 (59.0%)	7621 (63.1%)	903 (38.1%)	
Planned by a specialist	5656 (39.1%)	4218 (34.9%)	1439 (60.8%)	
Length of stay LOS, mean days ± SD				
LOS for total admission	9.3 ±13.1	9.0 ±13.0	11.0 ±13.2	0.001 ^#^
LOS outlier over DRG threshold (*n* = 725)	15.6 ± 24.0	16.2 ± 25.4	12.9 ± 14.7	0.153 ^#^
LOS outlier > 65 years old (*n* = 472)	14.6 ± 21.5	15.0 ± 22.4	12.4 ± 15.2	0.363 ^#^

# Student *t*-tests. * chi-square test.

**Table 2:** Association between cancer type and key variables affecting inpatient care (LOS outliers, infections, older age ≥ 65 years, and secondary diagnosis).

Type of Cancer	*n*°	Variable	Univariate OR (95% CI)	*p* Value	Multivariate AOR (95% CI) *	*p*-Value
Blood cancers	475	LOS outlier	2.658 (1.990–3.551)	<0.001	2.031 (1.499–2.753)	<0.001
		Age ≥ 65 years	0.664 (0.553–0.798)	<0.001	0.590 (0.489–0.711)	<0.001
		Infections	2.435 (1.988–2.983)	<0.001	2.368 (1.911–2.933)	<0.001
		Secondary diagnosis	1.310 (1.246–1.377)	<0.001	1.302 (1.176–1.858)	<0.001
Breast cancers	326	LOS outlier	0.290 (0.120–0.704)	0.006		NS
		Age ≥ 65 years	0.479 (0.382–0.601)	<0.001	0.516 (0.412–0.648)	<0.001
		Infections	0.156 (0.080–0.303)	<0.001	0.173 (0.089–0.336)	<0.001
		Secondary diagnosis	0.667 (0.620–0.717)	<0.001	0.716 (0.662–0.773)	<0.001
Nervous system cancers	323	LOS outlier	1.537 (1.007–2.346)	0.046	1.940 (1.255–2.998)	0.003
		Age ≥ 65 years	0.691 (0.554–0.862)	0.001	0.711 (0.570–0.889)	0.003
		Infections	0.547 (0.373–0.803)	0.002	0.517 (0.348–0.768)	0.001
		Secondary diagnosis	0.857 (0.828–0.932)	<0.001	0.906 (0.848–0.967)	0.003
Gastric cancers	183	LOS outlier	1.705 (1.000–2.907)	0.050		NS
		Age ≥ 65 years	1.813 (1.322–2.487)	<0.001	1.673 (1.216–2.301)	0.002
		Infections	2.382 (1.728–3.283)	<0.001	2.216 (1.603–3.062)	<0.001
		Secondary diagnosis	1.637 (1.486–1.802)	<0.001	1.637 (1.486–1.802)	<0.001
Hepatopancreatic cancers	487	LOS outlier	0.479 (0.263–0.837)	0.010	0.460 (0.258–0.820)	0.008
		Age ≥ 65 years	1.228 (1.021–1.477)	0.029	1.241 (1.032–1.494)	0.022
		Infections		NS		NS
		Secondary diagnosis	1.485 (1.407–1.568)	<0.001	0.382 (0.213–0.686)	<0.001
Lung cancers	345	LOS outlier		NS		NS
		Age ≥ 65 years	1.814 (1.440–2.286)	<0.001	1.862 (1.476–2.348)	<0.001
		Infections		NS	0.689 (0.495–0.959)	0.027
		Secondary diagnosis		NS		NS
Bladder Cancers	203	LOS outlier		NS		NS
		Age ≥ 65 years	2.827 (2.269–3.521)	<0.001	2.661 (2.133–3.319)	<0.001
		Infections	2.156 (1.749–2.657)	<0.001	1.905 (1.543–2.352)	<0.001
		Secondary diagnosis	1.129 (1.051–1.213)	0.001		NS

* The multivariate model included all the variables listed in the table (LOS outlier, age ≥ 65 years, and infections) simultaneously. No additional covariates were included in the model beyond those presented. NS = not significant.

**Table 3:** Association between different types of discharge in cancer-related hospitalizations.

Type of Discharge	*n*° of Discharges	LOS in Days Mean ± SD	Univariate OR (95% CI)	*p*-Value	Multivariate AOR (95% CI) *	*p*-Value
Home without activation of home care services	1110	12.3 ± 14.4	0.698 (0.639–0.762)	<0.001		NS
Home awaiting the completion of the diagnostic/therapeutic process	508	5 ± 6.7	1.997 (1.785–2.235)	<0.001	2.150 (1.911–2.418)	<0.001
Protected discharge	406	8.2± 7.9	1.601 (1.419–1.806)	<0.001	1.805 (1.591–2.047)	<0.001
Death	158	18.5 ± 16.6	1.237 (1.034–1.481)	0.020	1.472 (1.225–1.768)	<0.001
Territory residential facility	90	18.8 ± 13.7	0.685 (0.547–0.858)	0.001		NS
Transfer to post-acute /rehabilitation facility	44	22.6 ± 15.7	0.298 (0.219–0.405)	<0.001	0.375 (0.275–0.511)	<0.001
Voluntary	31	9.7 ± 11.2		NS		0.438
Acute care hospital	15	22.3 ± 22.6	0.297 (0.176–0.501)	<0.001	0.363 (0.215–0.613)	<0.001
Home with activation of home care service	6	34.3 ± 29.1		NS		0.724

* The multivariate model included all the variables listed in the table (LOS outlier, age ≥ 65 years, and infections) simultaneously. No additional covariates were included in the model beyond those presented. NS = Not significant.

## Data Availability

The data presented in this study are available on request from the corresponding author due to privacy reasons.

## Abbreviations

The following abbreviations are used in this manuscript:

AOR	Adjusted Odds Ratio
CEI	Independent Ethics Committee
CI	Confidence Intervals
DRGs	Diagnosis-Related Groups
GDP	Gross domestic product
ICD-9	International Classification of Diseases, 9th revision
LOS	Length of stay
NHF	National Health Fund
OR	Odds Ratio
PTV	Policlinico Tor Vergata
SDO	Scheda di Dimissione Ospedaliera
SPSS	Statistical Package for the Social Sciences

## References

[1] World Health Organization Cancer. https://www.who.int/news-room/fact-sheets/detail/cancer.

[2] Numico G., Zanelli C., Ippoliti R., Rossi M., Traverso E., Antonuzzo A., Bellini R. (2020). The hospital care of patients with cancer: A retrospective analysis of the characteristics of their hospital stay in comparison with other medical conditions. Eur. J. Cancer.

[3] Luengo-Fernandez R., Leal J., Gray A., Sullivan R. (2013). Economic burden of cancer across the European Union: A population-based cost analysis. Lancet Oncol..

[4] Sullivan R., Peppercorn J., Sikora K., Zalcberg J., Meropol N.J., Amir E., Khayat D., Boyle P., Autier P., Tannock I.F. (2011). Delivering affordable cancer care in high-income countries. Lancet Oncol..

[5] Statista Research Department Percentage of Elderly Population in Italy from 2009 to 2024. https://www.statista.com/statistics/785104/elderly-population-in-italy/.

[6] Longo F., Locatelli F., Del Vecchio M., Di Giulio P., Giordano S., Odone A., Ranieri V.M., Vineis P. (2024). Tackling the crisis of the Italian National Health Fund. Lancet Public Health.

[7] Eurostat Hospital Discharges and Length of Stay Statistics. https://ec.europa.eu/eurostat/statistics-explained/index.php?title=Hospital_discharges_and_length_of_stay_statistics&oldid=540628.

[8] Stone K., Zwiggelaar R., Jones P., Mac Parthaláin N. (2022). A systematic review of the prediction of hospital length of stay: Towards a unified framework. PLoS Digit. Health.

[9] Trevisan C., Noale M., Zatti G., Vetrano D.L., Maggi S., Sergi G. (2023). Hospital length of stay and 30-day readmissions in older people: Their association in a 20-year cohort study in Italy. BMC Geriatr..

[10] Carestia M., Andreoni M., Buonomo E., Ciccacci F., De Angelis L., De Carolis G., De Filippis P., Di Giovanni D., Emberti Gialloreti L., Fontana C. (2023). A novel, integrated approach for understanding and investigating Healthcare Associated Infections: A risk factors constellation analysis. PLoS ONE.

[11] Giraldi G., Montesano M., Napoli C., Frati P., La Russa R., Santurro A., Scopetti M., Orsi G.B. (2019). Healthcare-Associated Infections Due to Multidrug-Resistant Organisms: A Surveillance Study on Extra Hospital Stay and Direct Costs. Curr. Pharm. Biotechnol..

[12] Ministero Della Salute Rapporto Annuale Sull’attività di Ricovero Ospedaliero: Dati SDO 2019. https://www.salute.gov.it/imgs/C_17_pubblicazioni_3002_allegato.pdf.

[13] FADOI Ospedali, Anziani Dimessi 7 Giorni più Tardi. Oltre 2 Giornate di Degenza Improprie. L’indagine FADOI. https://www.fadoi.org/press-room/ospedali-anziani-dimessi-7-giorni-piu-tardi-oltre-2-giornate-di-degenza-improprie-lindagine-fadoi/?_gl=1*1sb1iyz*_up*MQ..*_ga*MTk0MzYzMDg5Ni4xNzE4NjYwOTg3*_ga_CBP79GC2JJ*MTcxODY2MDk4Ny4xLjEuMTcxODY2MTE1Mi4wLjAuMA.

[14] Wang R.C., Wang Z. (2023). Precision Medicine: Disease Subtyping and Tailored Treatment. Cancers.

[15] Greenberg J.A., David M.Z., Pitrak D.L., Hall J.B., Kress J.P. (2014). Prior infections are associated with increased mortality from subsequent blood-stream infections among patients with hematological malignancies. Eur. J. Clin. Microbiol. Infect Dis..

[16] Waguia R., Wang T.Y., Mehta V.A., Ramirez L., McCray E., Pennington Z., Price M., Dalton T., Baëta C., Sciubba D.M. (2021). Risk factors for prolonged length of stay in patients undergoing surgery for intramedullary spinal cord tumors. J. Clin. Neurosci..

[17] Regione Lazio (2020). DE G16239 del 24.12.2020 Rete e PDTA Mammella. https://www.regione.lazio.it/sites/default/files/documentazione/2024/DE%20G16239%20del%2024.12.2020%20Rete%20e%20PDTA%20Mammella.pdf.

[18] Pîslaru A.I., Albișteanu S.-M., Ilie A.C., Ștefaniu R., Mârza A., Moscaliuc Ș., Nicoară M., Turcu A.-M., Grigoraș G., Alexa I.D. (2024). Lung Cancer: New Directions in Senior Patients Assessment. Geriatrics.

[19] Higuera O., Ghanem I., Nasimi R., Prieto I., Koren L., Feliu J. (2016). Management of pancreatic cancer in the elderly. World J. Gastroenterol..

[20] Cho E., Cho H.A., Jun C.H., Kim H.J., Cho S.B., Choi S.K. (2019). A Review of Hepatocellular Carcinoma in Elderly Patients Focused on Management and Outcomes. In Vivo.

[21] Gentile L., Scaramella M., Liotta G., Magrini A., Mulas M.F., Quintavalle G., Palombi L. (2024). Limitations and consequences of public health models centred on hospitals and lacking connections with territorial and home-based social and health services. Int. J. Emerg. Med..

[22] Legramante J.M., Morciano L., Lucaroni F., Gilardi F., Caredda E., Pesaresi A., Coscia M., Orlando S., Brandi A., Giovagnoli G. (2016). Frequent Use of Emergency Departments by the Elderly Population When Continuing Care Is Not Well Established. PLoS ONE.

[23] Gigantesco A., de Girolamo G., Santone G., Miglio R., Picardi A., PROGRES-Acute group (2009). Long-stay in short-stay inpatient facilities: Risk factors and barriers to discharge. BMC Public Health.

[24] Hoogervorst-Schilp J., Langelaan M., Spreeuwenberg P., de Bruijne M.C., Wagner C. (2015). Excess length of stay and economic consequences of adverse events in Dutch hospital patients. BMC Health Serv. Res..

[25] Glasziou P., Straus S., Brownlee S., Trevena L., Dans L., Guyatt G., Elshaug A.G., Janett R., Saini V. (2017). Evidence for underuse of effective medical services around the world. Lancet.

[26] Mennini F.S., Marcellusi A., Sciattella P., Scortichini M., Ragonese A., Cattel F., D’Antona R., Del Mastro L., Gori S., Perrone G. (2025). Burden of Disease of Breast Cancer in Italy: A Real-World Data Analysis. Pharmacoecon Open.

[27] Hanan T., Mullen L., Laffoy M., O’Toole E., Richmond J., Wynne M. (2014). Delivering care to oncology patients in the community: An innovative integrated approach. Br. J. Community Nurs..

[28] Liljas A.E.M., Brattström F., Burström B., Schön P., Agerholm J. (2019). Impact of Integrated Care on Patient-Related Outcomes Among Older People—A Systematic Review. Int. J. Integr. Care.

[29] Damery S., Flanagan S., Combes G. (2016). Does integrated care reduce hospital activity for patients with chronic diseases? An umbrella review of systematic reviews. BMJ Open.

[30] World Health Organization Regional Office for the Eastern Mediterranean. The Growing Need for Home Health Care for the Elderly. https://iris.who.int/bitstream/handle/10665/326801/EMROPUB_2015_EN_1901.pdf?sequence=1&isAllowed=y.

[31] Allan S., Roland D., Malisauskaite G., Jones K., Baxter K., Gridley K., Birks Y. (2021). The influence of home care supply on delayed discharges from hospital in England. BMC Health Serv. Res..

[32] NHS England and NHS Improvement (2021). Building Strong Integrated Care Systems Everywhere ICS Implementation Guidance on Working with People and Communities London, England. https://www.england.nhs.uk/wp-content/uploads/2021/06/B0661-ics-working-with-people-and-communities.pdf.

[33] Wiernik A., Rogado A., O’Mahony D., Abdul Razak A.R. (2024). Elevating Cancer Care Standards Worldwide: An Analysis of Global Initiatives and Progress. JCO Glob. Oncol..

[34] Mariotti S., Minuti G., Landi L., Bria E., Carlucci G., Di Salvatore M., Giusti R., Iurato A., Ramella S., Ricciotti M.A. (2024). Simultaneous care provision to patients with small cell lung cancer in Lazio region: Practical recommendations of a multidisciplinary group. Heliyon.

[35] OECD/European Commission (2025). EU Country Cancer Profile: Italy 2025, EU Country Cancer Profiles.

[36] Bidoli C., Pegoraro V., Dal Mas F., Bagnoli C., Cordiano C., Minto G., Zantedeschi M., Stocco P., Bonin M., Pilerci C. (2022). Virtual hospital: Il futuro del sistema sociosanitario? Un approccio basato su un expert consensus all’interno della Regione Veneto. Politiche Sanit..

[37] Wong A., Cooper C., Evans C.J., Rawle M.J., Walters K., Conroy S.P., Davies N. (2025). Supporting older people through Hospital at Home care: A systematic review of patient, carer and healthcare professionals’ perspectives. Age Ageing.

[38] Dambha-Miller H., Simpson G., Hobson L., Olaniyan D., Hodgson S., Roderick P., Fraser S.D., Little P., Everitt H., Santer M. (2021). Integrating primary care and social services for older adults with multimorbidity: A qualitative study. Br. J. Gen. Pract..

[39] Istituto Superiore di Sanità (2023). EpiCentro—L’epidemiologia per la Sanità Pubblica. Ricoveri Ospedalieri in Italia: Il Rapporto SDO. https://www.epicentro.iss.it/sdo/rapporto-sdo-2023.

